# CTSB promotes sepsis-induced acute kidney injury through activating mitochondrial apoptosis pathway

**DOI:** 10.3389/fimmu.2022.1053754

**Published:** 2023-01-13

**Authors:** Yuting Wang, Wenjie Xi, Xinyi Zhang, Xinwen Bi, Boyang Liu, Xiaoming Zheng, Xinjin Chi

**Affiliations:** ^1^ Department of Anesthesiology, The Seventh Affiliated Hospital of Sun Yat-sen University, Shenzhen, China; ^2^ Scientific Research Center, The Seventh Affiliated Hospital of Sun Yat-sen University, Shenzhen, China

**Keywords:** lysosomal membrane permeabilization, CA074, mitochondrial apoptosis pathway, S-AKI, cathepsin B (CTSB)

## Abstract

**Background:**

Acute kidney injury is a common and severe complication of sepsis. Sepsis -induced acute kidney injury(S-AKI) is an independent risk factor for mortality among sepsis patients. However, the mechanisms of S-AKI are complex and poorly understand. Therefore, exploring the underlying mechanisms of S-AKI may lead to the development of therapeutic targets.

**Method:**

A model of S-AKI was established in male C57BL/6 mice using cecal ligation and puncture (CLP). The data-independent acquisition (DIA)-mass spectrometry-based proteomics was used to explore the protein expression changes and analyze the key proteomics profile in control and CLP group. The methodology was also used to identify the key proteins and pathways. S-AKI *in vitro* was established by treating the HK-2 cells with lipopolysaccharide (LPS). Subsequently, the effect and mechanism of Cathepsin B (CTSB) in inducing apoptosis in HK-2 cells were observed and verified.

**Results:**

The renal injury scores, serum creatinine, blood urea nitrogen, and kidney injury molecule 1 were higher in septic mice than in non-septic mice. The proteomic analysis identified a total of 449 differentially expressed proteins (DEPs). GO and KEGG analysis showed that DEPs were mostly enriched in lysosomal-related cell structures and pathways. CTSB and MAPK were identified as key proteins in S-AKI. Electron microscopy observed enlarged lysosomes, swelled and ruptured mitochondria, and cytoplasmic vacuolization in CLP group. TUNEL staining and CTSB activity test showed that the apoptosis and CTSB activity were higher in CLP group than in control group. In HK-2 cell injury model, the CTSB activity and mRNA expression were increased in LPS-treated cells. Acridine orange staining showed that LPS caused lysosomal membrane permeabilization (LMP). CA074 as an inhibitor of CTSB could effectively inhibit CTSB activity. CCK8 and Annexin V/PI staining results indicated that CA074 reversed LPS-induced apoptosis of HK-2 cells. The JC-1 and western blot results showed that LPS inhibited mitochondrial membrane potential and activated mitochondrial apoptosis pathway, which could be reversed by CA074.

**Conclusions:**

LMP and CTSB contribute to pathogenesis of S-AKI. LPS treatment induced HK-2 cell injury by activating mitochondrial apoptosis pathway. Inhibition of CTSB might be a new therapeutic strategy to alleviate sepsis-induced acute kidney injury.

## 1 Introduction

Sepsis is a life-threatening organ dysfunction resulting from a dysregulated inflammatory to infection (1, 2). If not recognized and treated early, it could develop into septic shock, multiple organ failure and death. Data from the cause-of-death surveillance system database involving 323.8 million people showed that sepsis-related deaths accounted for 12.6% of deaths (3). The kidney is highly perfused, making it more sensitive and vulnerable to sepsis. Acute kidney injury (AKI) is one of the most common and severe complications of sepsis, resulting in prolonged hospitalization and a poor prognosis (4).

Although sepsis is an important cause of AKI in critically ill patients, the pathophysiological mechanisms of S-AKI are still poorly understood,. However, the potential mechanisms may include altering renal blood flow, microcirculatory disturbances, over-activated immune-inflammatory responses and metabolic reprogramming (5–7). The most prominent pathological changes resulting AKI are the injury and death of tubular epithelial cells. Acute renal injury is characterized by apoptosis and necrosis in renal cells or tubule (8, 9). Cells affected by acute necroinflammation during sepsis are irreversibly lost and replaced by fibrous tissue and finally affecting renal function. Tubule apoptosis and necrosis in AKI result in significant nephron loss. The number of irreversibly damaged nephrons determines the functional capacity of the kidney. Hence, it is crucial to develop strategies to prevent renal cell death and reduce irreversible damage to the nephrons in the context of sepsis.

Lysosomes were first described in 1955 by Christian de Duve (10). Lysosomes are monolayer membrane-enclosed organelles containing about 60 hydrolases, including cathepsins, ceramidases, and patatins. The main function of lysosome is to degrade and recycle extracellular substances transported by pinocytosis and intracellular substances such as damaged organelles or misfolded proteins transported by autophagy. In addition, growing evidence suggests that lysosomes are also an important signaling hub that regulate multicellular processes, including energy metabolism, signal transmission, and cell death regulation (11). Lysosomes are involved in several cell death pathways (12, 13). The most common lysosome-induced cell death pathway is lysosome-dependent cell death (LDCD), characterized by lysosomal destabilization and requiring lysosomal membrane permeabilization (LMP). LDCD is mediated by cathepsins and other hydrolases released into the cytoplasm from damaged lysosomes. LMP and the release of cathepsins initiate apoptosis by inducing the cleavage of caspases protein family and Bcl-2 protein family (14, 15). Since the kidney is a lysosome-rich organ, LMP and cathepsins are observed in many renal diseases. Lysosomal dysfunction and cathepsins have been detected in renal tubular epithelial cells in different AKI animal models, such as nephrotoxic injury and ischemia-reperfusion injury (16, 17).

Cathepsin B (CTSB) is a cysteine proteolytic enzyme found in the lysosomes. Increased CTSB activity is considered an indicator of LMP. Studies have confirmed that CTSB is a risk factor for tumor cell migration, proliferation, and apoptosis and synchronously affects cancer angiogenesis and chemoresistance (18, 19). The CTSB levels have been found to influence aging-related renal subclinical alterations in healthy populations and are independently correlated with renal function in elderly women (20). Lysosomes and cathepsins have been widely proven to be involved in cell injury and death pathways, including LDCD, lysosomal-mitochondrial apoptosis, and autophagy-lysosomal pathways (21).

However, the specific role of LMP and CTSB in S-AKI is not known and needs to be explored. In this study, we established an S-AKI model in mice to explore the changes of protein expression in renal tissues and identify potential key molecules and mechanisms through proteomic analysis. *In vitro* experiments were conducted to investigate and verify the role of LMP and CTSB.

## 2 Materials and methods

### 2.1 Animals and S-AKI model

Male C57BL/6 mice were purchased from Guangdong yaokang biology technology company at 8-10 weeks old age, weighing 20-25 g. Animal Ethics Committee approved all animal procedures (SYSU-IACUC-2021-00559). The mice were randomly divided into the control group and CLP group. In the CLP group, the sepsis models were induced by cecal ligation and puncture (CLP) and the mice were sacrificed at 12 h after CLP surgery. The blood and kidney tissues were collected for subsequent experiments.

### 2.2 Histological assessment of kidney tissue

Hematoxylin and eosin (H&E) staining was conducted according to routine protocols. Slides were visualized using a microscopy. Kidney tubule injury was defined as loss of brush border, vacuolar degeneration, and cast formation (22). According to the percentage of injury area, the scores of renal pathological damages were completed by 2 independent pathologists in the department of pathology of our hospital.

### 2.3 Data independent acquisition (DIA) analysis and proteomics analysis

The proteomics experiments were carried out by the HuaDa company (HuaDa, China) and bioinformatics analysis was performed by Dr.TOM on-line system software developed by the Beijing Genomics Institute (http://report.bgi.com). The renal tissues were lysed and total protein was extracted. Bradford was used to quantify and draw the standard curve of protein concentration, and the protein quality was evaluated. Protein identification and analysis completed by MaxQuant (UniProt protein database). Acquire all detectable non-redundant MS/MS spectrogram information, including retention characteristics, peak intensities, and integrated mass spectra. The FDR was set to ≤ 1% for identification of both proteins and peptides. Data dependent acquisition (DDA) was used to build a spectra library for DIA analysis. Extracted ion chromatograms were generated and peak areas were obtained by integration. The MSstats R package was used for normalization and protein quantification. The Fold change > 2 and adj *p*-value <0.05(Q-value) were used as the screening criteria for proteins with significant differences. After the differentially expressed proteins (DEPs) between the control group and CLP group were obtained, the proteins were annotated by GO function annotation and KEGG pathway enrichment analysis. Gene Ontology (GO) identifies three aspects of biology: cellular component (CC), biological process (BP), and molecular function (MF). The dataset related to apoptosis was extracted from KEGG database, and the gene-coding proteins were obtained by STRING (https://cn.string-db.org/). The intersection analysis of DEPs and apoptosis-related proteins in control and CLP groups was carried out to obtain the key proteins of S-AKI.

### 2.4 Luminex assay

Blood samples were obtained from eyeballs. Serum creatinine, blood urea nitrogen (BUN), and kidney injury molecule 1 (KIM-1) were measured using Luminex assay (Mouse Premixed Muti-Analyte Kit, LXSAMSM-05). The Luminex assay was performed with Luminex LX100 system, which allows for simultaneous measurements of multiple analytes. Each measurement was performed in pg/ml on the Luminex platform, and the results were expressed as median fluorescence intensity.

### 2.5 TUNEL staining

TUNEL Apoptosis Assay kit (Beyotime, China) was used to measure apoptotic cells of renal tissue. The sections were deparaffinized and treated with Proteinase K for 30 min at 37°C. After washing with PBS twice, the slides were incubated with TUNEL detection solution for 1 h. The red fluorescence of apoptotic cells was captured by a fluorescent microscope (Ex/Em = 550/570 nm). Apoptotic cells were counted in three randomly selected fields.

### 2.6 Cathepsin B activity

Cathepsin B activity was measured using fluorometric Cathepsin B Activity Assay (Abcam, USA). Tissue samples and cell samples were prepared following the manufacturer’s protocol. 50 μL samples and 50 μL reaction buffer were added to black 96-well plates. 2 μL CTSB substrate (Ac-RR-AFC; 200 μM final concentration) was added and the mixture was incubated for 1 h at 37°C. Measure output on a fluorescent microplate reader at Ex/Em = 400/505 nm.

### 2.7 Transmission electron microscopy (TEM)

The morphological changes of kidney tissues were observed by TEM. Kidney tissues were cut into about 1 mm^3^ pieces and placed in 4% glutaraldehyde at 4°C for 12 h. TEM was performed by Wuhan Servicebio Technology company. Hitachi HT7700 TEM (Hitachi, Japan) was used for imaging ultrastructure.

### 2.8 Cell culture and treatment

Human proximal tubular epithelial cells (HK-2) were obtained from American Type Cell Culture (ATCC). Cells were cultured in DMFM/F-12 (Gibco, USA) containing 10% fetal bovine serum in a cell incubator with 5% CO_2_ maintained at 37°C. HK-2 cells were treated with different concentrations of LPS for 24 h or 48 h. CTSB inhibitor CA074 was purchased from MERCK (134448-10-5). HK-2 cells were treated with 5 μM CA074.

### 2.9 Cytotoxicity assay

Cytotoxicity assay was measured by the Cell Counting Kit 8 assay (CCK8) and Annexin V-FITC/PI Apoptosis Detection Kit. Cell viability was measured with CCK8 (Yeasen, China). HK-2 cells (4 × 10^3^ cells/well) were cultivated in 96-well plates. LPS was added to plates. At the appropriated time point, 10 μL of CCK8 was added to each well and incubated for 3 h. The results were assayed with a wavelength of 450 nm using a microplate reader. An Annexin V-FITC/PI Apoptosis Detection Kit (Beyotime, China) was used to assess cell apoptosis. Cells were washed with cold PBS for 2 times and were harvested by trypsin without EDTA. Cells were suspended in 195 μL binding solution. Then, Annexin V-FITC (5 μL) and propidium iodide (10 μL) staining solution were added and incubated in an ice bath avoiding light for 15 min. The ratio of apoptotic cells was determined by flow cytometry.

### 2.10 Lysosome membrane permeability (LMP)

Acridine orange (AO) (sigma, USA) was performed to assess the LMP. HK-2 cells were incubated by AO (5 μg/mL) for 20 min at 37°C. Cells were visualized using a confocal scanning microscope. The excitation wavelength was 488 nm, while the data were obtained at two separate emission wavelengths (505-560 nm, 590-690 nm). The decreased red fluorescence and enhanced green fluorescence were the sign of LMP.

### 2.11 Mitochondrial membrane potential (MMP)

MMP was assessed using JC-1 kit (Beyotime, China). Cells were incubated with JC-1 working solution for 20 min at 37°C in the dark. Cells were washed with cold JC-1 staining buffer for 2 times, and observed by fluorescence microscopy. Red J-aggregate emission (Ex/Em=525/590 nm) was observed in normal cells, and JC-1 monomers (Ex/Em=490/530 nm) were generated as membrane potential decreased, resulting in green emission.

### 2.12 Real-time PCR

The total RNA was extracted from HK-2 cells with TRIzol reagent, and the cDNA is synthesized using PrimeScript™ RT reagent Kit (Takara, Japan). Real-time PCR was performed using SYBR Green Real-time PCR master mix on DNA Engine Opticon 2 System. The PCR cycle is 95 °C for 10 seconds, 60 °C for 30 seconds. The expression levels of CTSB and GAPDH were assessed using the 2^−ΔΔCt^ method. The primers used were as follows: CTSB: F: CGCGGCTCAAAAGGAAACC; R: TTAACTTGACAG GGTGAAGCTG. GAPDH: F: TGTTGCCATCAATGACCCCTT; R: CTCCACG ACGTACTCAGCG.

### 2.13 Western blot

The total protein concentration was determined by BCA assay (Beyotime, China). And the protein concentrations were adjusted to 1μg/μL. Cell lysates were boiled at 100°C for 5 min, separated on 12% SDS–PAGE and transferred to 0.22 μm PVDF membrane. Membranes were blocked with 5% non-fat milk at room temperature for 1.5 h. Then, the membranes were incubated with the primary antibodies overnight at 4 °C, followed by incubation with the secondary antibodies. Immunoblots were visualized using ECL and analyzed using Image J software. The following antibodies (Abclonal, China) were used: anti-β-actin (AC006), anti-caspase3(A19654), anti-PARP (A11010), anti-Bax (A12009), anti-Bcl2(A19693) and HRP Goat Anti-Rabbit IgG (H+L) (AS014).

### 2.14 Statistical analysis

Statistical analysis was performed by GraphPad Prism 8.0 software. Data were shown as means ± sem. The difference between the two groups was determined using Student t-test, and the difference among multiple groups was analyzed by the one-way analysis of variance, with *p* < 0.05.

## 3 Results

### 3.1 Established sepsis-induced AKI model in mice

**Figure 1:**
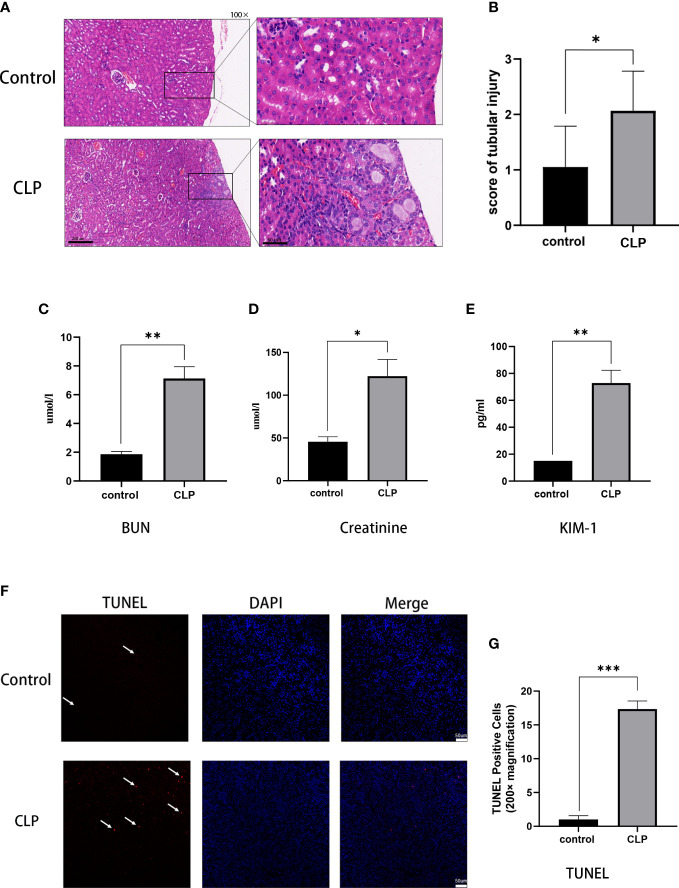
S-AKI model of renal injury in mice. **(A)** Representative histological images from HE staining kidney sections. Magnification, 100 x, 400 x. **(B)** Renal injury scores assessed based on evaluation of HE staining. **(C)** Serum BUN, **(D)** serum creatinine, **(E)** serum KIM-1 as measures of kidney function. **(F)** Images of TUNEL staining, red: TUNEL-positive cells, blue: DAPI. **(G)** TUNEL-positive cells rat. Magnification, 200x. Data expressed as mean ± sem. *p < 0.05, **p < 0.01, ***p < 0.001, ****p < 0.0001 vs. control group.

Renal histopathological analysis and renal function tests were performed to evaluate kidney injury. HE staining showed that the renal structure in the control group was intact with no sign of swelling, denaturation, necrosis, and inflammatory cell infiltration. The pathological changes in the kidney tissue were aggravated over the 12 h post-CLP. Renal tubular epithelial cell swelling, necrosis, vacuolar degeneration, and renal tubular lumen expansion were observed in the CLP group (**Figure 1A**). The renal tubular injury scores were elevated (**Figure 1B**). Renal function and injury biomarkers were evaluated using the serum creatinine, BUN, and KIM-1(**Figures 1C-E**). The creatinine and BUN levels were about 3-fold higher in the CLP group than in the control group. In addition, KIM-1 was higher in CLP mice than in the control group. TUNEL staining was used to assess apoptosis in kidney tissues. Few apoptotic cells were found in the control group, and the number of TUNEL-positive cells in CLP group was significantly increased compared with control group(**Figures 1F, G**).

### 3.2 Proteomics analysis of renal tissue and identify CTSB as the key protein

**Figure 2:**
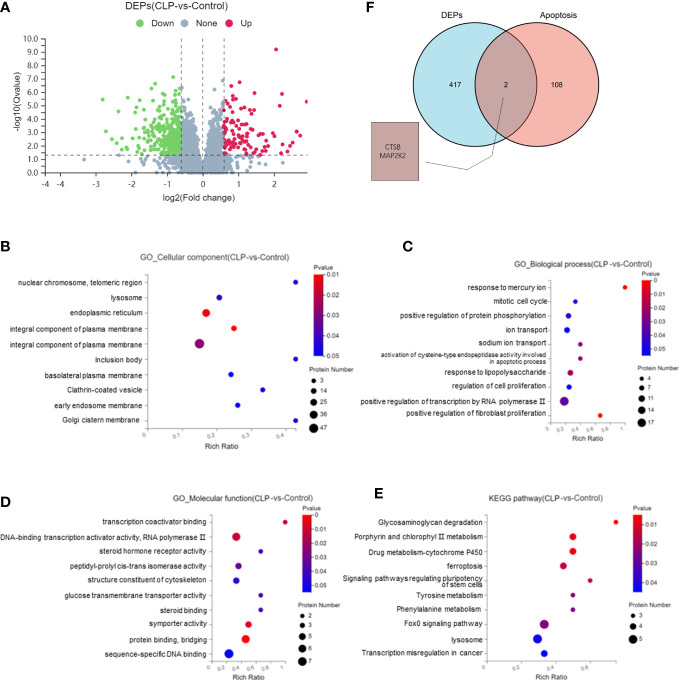
Proteomic analysis of renal tissues. **(A)** Volcano plot of differentially expressed proteins (DEPs). Bubble map of GO and KEGG pathway analysis: **(B)** Cellular Components, **(C)** Biological Process, **(D)** Molecular Function, **(E)** KEGG pathway. **(F)** Venn diagram of DEPs and apoptosis-related proteins.

The proteomics analysis was used to analyze renal tissues in the S-AKI model. The proteomics analysis analyzed six samples (three samples for each group) and identified 115,664 peptides and 21,805 proteins. Of the total proteins,11,054 proteins were found in the control group and 10,751 proteins in the CLP group. Compared with the control group, there were 108 upregulated proteins and 341 downregulated proteins in the CLP group (**Figure 2A**). GO analysis of the 449 DEPs was performed. In the CC category, the top GO terms were integral component of membrane, endoplasmic reticulum, and lysosome. In the BP category, the top GO terms were positive regulation of fibroblast proliferation, response of lipopolysaccharide, and ion transport. In the MF category, the top GO terms were DNA-binding transcription activator activity, sequence-specific DNA binding, and protein binding. KEGG pathway enrichment analysis showed that the lysosome, FoxO signaling pathway, and ferroptosis were the top 3 pathways with the most protein enrichment (**Figures 2B-E**). The intersection between DEPs and apoptosis-related proteins extracted from KEGG was analyzed. CTSB and MAP2K2 were selected as key proteins (**Figure 2F**). The functional annotation and pathway enrichment analysis suggested that lysosome was the key cell component and pathway in S-AKI. In addition, the result showed that CTSB is one of the most important cathepsins in the lysosome. The proteomic analysis illustrated that lysosomes and CTSB might be the key drivers of S-AKI development.

### 3.3 Lysosomal membrane permeabilization and CTSB activity in S-AKI

**Figure 3:**
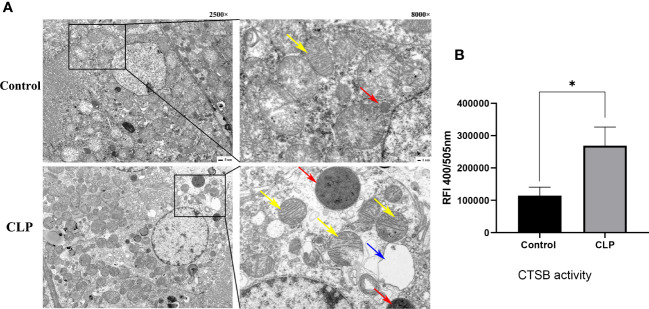
Lysosome and CTSB. **(A)** Transmission electron microscope (TEM) images. Magnification, 2500 x, 8000 x. Red arrows: lysosome, yellow arrows: mitochondria, blue arrows: cellular vacuolization. **(B)** The CTSB activity of renal tissue. Data expressed as mean ± sem. *p < 0.05, vs. control group.

The ultrastructural changes in renal tissue were observed by TEM. In the control group, the nuclei and organelles had normal structures. A single layer of membrane was observed in lysosomes, which contained numerous internal substances. The TEM revealed that intracellular vacuoles were formed, mitochondria were swollen and rounded, the number and volume of lysosomes were increased in CLP renal tissues. The increased size of lysosomes suggested that lysosomes were dysfunctional. Cytoplasmic vacuoles and mitochondria swelling and rupture indicated the apoptosis occurred in renal tissue (**Figure 3A**). Analysis of the activity of CTSB found that the activity of CTSB was higher in the CLP group than in the control group (**Figure 3B**). The data above suggested that lysosomes and CTSB were involved in S-AKI, and LMP and CTSB leakage were associated with kidney injury in sepsis.

### 3.4 LPS increased the CTSB activity and LMP in HK-2 cells

**Figure 4:**
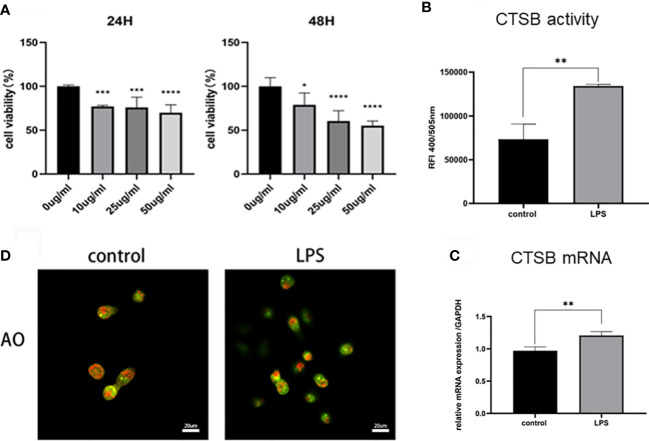
LPS-induced HK-2 cell injury model. **(A)** Cell viability rate of different concentrations of LPS using the CCK8 assay. **(B)** CTSB activity and **(C)** mRNA expression in HK-2 cells. **(D)** Lysosomal membrane stability by AO staining under confocal microscope. Magnification,1000 x. Data expressed as mean ± SD. *p < 0.05, **p < 0.01, ***p < 0.001, ****p < 0.0001vs. control group.

To verify the hypothesis, we further established an LPS-induced HK-2 cell injury model to investigate the role of lysosomes and CTSB. HK-2 cells were treated with different concentration of LPS (10 μg/mL, 25 μg/mL, 50 μg/mL) for 24 h, 48 h and subjected to the CCK8 assay. The results showed that LPS inhibited the growth of HK-2 cells significantly, 10 μg/mL LPS treated for 24 h was the optimum concentration of LPS and was thus selected for further experiments (**Figure 4A**). Subsequently, we stimulated HK-2 cells with LPS and the CTSB activity and mRNA expression were investigated. The results showed that LPS induced a significant increase in the activity and mRNA expression of CTSB (**Figures 4B, C**). To confirm lysosomal permeability, the lysosomes were stained with the OA staining,and observed under a confocal microscope. After LPS stimulation, the red particles obviously decreased which indicated an increase in lysosomal permeability (**Figure 4D**). The above results suggested that LMP occurred and CTSB activity and mRNA expression increased in the LPS-induced HK-2 injury cell model

### 3.5 Inhibition of CTSB activity alleviated LPS-induced HK-2 apoptosis

**Figure 5:**
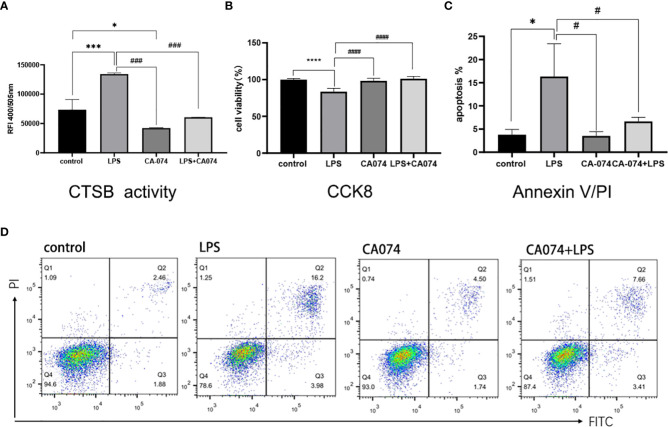
CA074 inhibited CTSB activity and relieved LPS-induced HK-2 apoptosis. **(A)** The influence of CA074 in CTSB activity. **(B)** The cell viability rate of HK-2 cells treated with CA074 using the CCK8 assay. **(C)** Flow cytometry analysis of apoptosis in HK-2 cells treated with CA074 by Annexin V/PI staining. **(D)** The percentage of apoptotic cells in different groups determined by flow cytometry analysis. Data expressed as mean ± SD. *p < 0.05, ***p < 0.001, ****p < 0.0001 vs. control group. *#p < 0.05, ###p < 0.001, ####p < 0.0001* vs. LPS group.

To determine whether inhibition the CTSB activity could alleviated LPS-induced cell injury in HK-2 cells, the CTSB inhibitor CA074 and CA074+LPS treatment were utilized Analysis of the CTSB activity results showed that compared with the control group, CA074 significantly inhibited the activity of CTSB(**Figure 5A**). HK-2 cells treated with LPS+CA074 showed higher cell viability rate than those treated with LPS. This indicated that CA074 could reverse the effect of LPS (**Figure 5B**). To further clarify the effect of CA074 against the LPS-induced apoptosis, Annexin V/PI double staining was performed. As shown in **Figures 5C, D**, the percentage of apoptotic cells was higher in LPS treated cells than that of in LPS+CA074 treated cells. Those findings demonstrated that CA074 could prevent LPS-induced apoptosis by inhibiting the increase in CTSB activity.

### 3.6 CTSB induced apoptosis by activating mitochondrial apoptosis pathway in HK-2 cells

**Figure 6:**
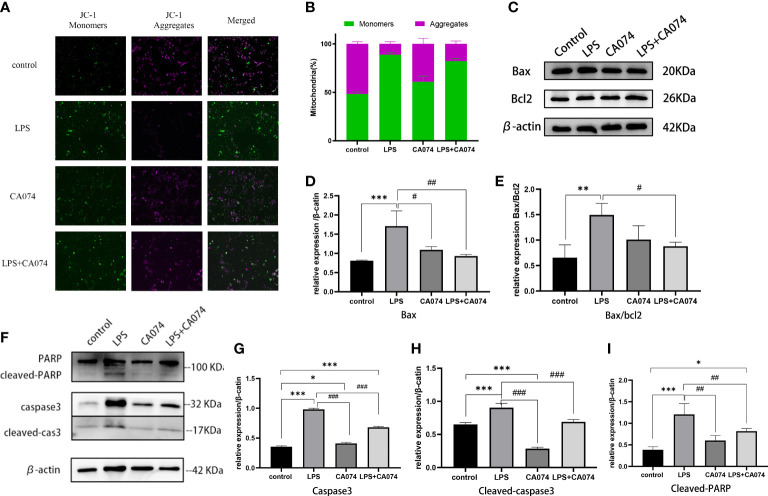
CTSB activate mitochondrial apoptosis pathway. **(A)** Representative images of JC-1 staining. Magnification,200 x. **(B)** The radio of JC‐1 green to red fluorescence. **(C)** Representative image of Bax and Bcl2 protein expression. **(D, E)** Western blotting analysis of Bax and Bax/bcl2. **(F)** Representative image of caspase3, cleaved-caspase3, PARP and cleaved-PARP protein expression. **(G-I)** Western blotting analysis of caspase3, cleaved-caspse3, cleaved-PARP. Data expressed as mean ± SD. *p < 0.05, **p < 0.01, ***p < 0.001 vs. control group. *#p < 0.05, ##p < 0.001, ###p < 0.001* vs. LPS group.

To investigate whether mitochondrial apoptosis pathway was involved in LPS-induced HK-2 cell injury. mitochondrial membrane potential was assessed. Using the membrane-permeant JC-1 dye, LPS-treated HK-2 cells showed a green fluorescence shift as the mitochondria became depolarized compared with control cells. A higher ratio of red-to-green fluorescence intensity ratio was observed after LPS treatment. The green fluorescence was significantly reversed in the cells co-treated with CA074+LPS (**Figures 6A, B**), confirming that CA074 could reverse the injury caused by CTSB. The effect of CTSB and CA074 on apoptosis-related proteins was measured with western blot. LPS treatment markedly increased the Bax/Bcl2 ratio (**Figures 6C-E**). In addition, the expression of cleaved-caspase3 and cleaved-PARP were higher in HK-2 cells compare with control group after LPS treatment (**Figures 6F-I**). In contrast, CA074 treatment decreased the expressions of Bax, cleaved-caspase3 and cleaved-PARP. These results suggested that CTSB inhibition could reverse apoptosis by blocking the Bax/Caspase3/PRAP activation.

## 4 Discussion

**Figure 7:**
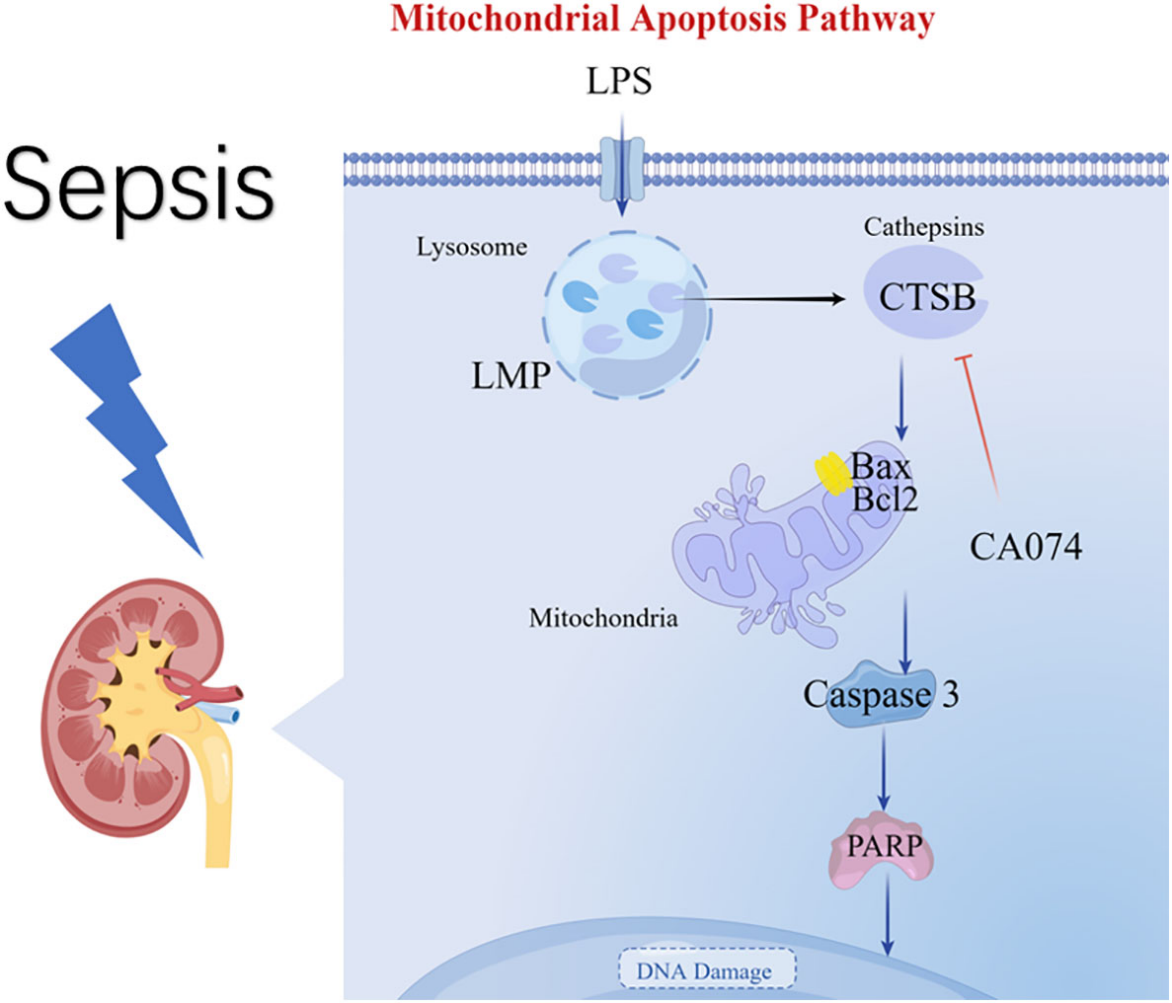
Diagram summarizing the key findings of our study. A model of S-AKI is established in mice, and proteomic analysis reveal that lysosomes and CTSB participate in the process of kidney injury. An *in vitro* experiment of LPS-induced HK-2 cell injury illustrate that LPS promotes LMP and leakage of CTSB, Changes in LMP and CTSB activity resulted in apoptosis, mitochondrial membrane potential changes, and activation of the Bax/caspase3/PARP proteins. CA074 can protect HK-2 cells from apoptosis by inhibition activity of CTSB.

In this study, the AKI model of sepsis in mice was established, and the protein spectrum detection and bioinformatics analysis of kidney tissues showed that lysosomes and CTSB exacerbate acute kidney injury in sepsis. Through *in vitro* experiments, we found that LMP and CTSB promoted apoptosis of HK-2 cells through the lysosome-mitochondrial axis. (**Figure 7**).

Proteins play important roles in various cellular activities. They can reflect the state of the body, and provide insights into the molecular changes (23, 24). Therefore We established a model of sepsis-induced renal injury in mice and performed protein spectrum detection and bioinformatics analysis to explore the mechanism underlying AKI. The GO CC analysis showed that the DEPs enriched in integral component of membrane and lysosome. The GO BP analysis showed that the DEPs enriched in response to lipopolysaccharide and apoptotic process. The results of KEGG analysis showed that lysosomes were enriched with proteins such as, Ap4m1, Gns, Hgsnat, Gusb, and Ctsb. To identify the key proteins, we explored the intersection of the DEPs with the apoptosis-related dataset, and found that the CTSB and MAP2K2 were the key proteins in S-AKI. Notably, CTSB, a proteolytic enzyme primarily found in lysosomes, is one of the indicators of LMP (25). And lysosomes were observed to swelling and becoming larger in the CLP group through TEM. Those findings confirmed that lysosomes participated in the development of S-AKI. TEM also observed the signs of cell apoptosis, including swelling and breaking of mitochondria and cell vacuolation. Furthermore, we found that the activity of CTSB was increased in CLP group, and the number of apoptotic cells exceeded that in the control group. Accordingly, it is reasonable to postulate that lysosome or LMP and CTSB pathogenesis of S-AKI.

Numerous studies have demonstrated that LMP and CTSB are involved in the pathogenesis of kidney diseases (26–29). For instance, abnormal lysosomal function and structure were found in diabetic kidney disease (DKD). Strategies that promoting lysosomal formation and maintaining the stability of lysosome membranes can alleviate glomerular podocytes and tubular epithelial cells injury and delay the progress of DKD (30). In the renal ischemia-reperfusion injury model, CTSB and CTSD were released from lysosome to cytoplasm, which activated NLRP3 and led to HK-2 cell injury (16). In the present study, our results showed for the first time that LMP and CTSB were involved in S-AKI.

An *in vitro* cell model was established to verify the hypothesis of LMP and CTSB. According to our results, LMP and leakage of CTSB resulted in apoptosis, mitochondrial membrane potential changes, increased ROS production, and activation of the Bax/caspase3/PARP proteins. Further, CA074, a highly selective inhibitor of CTSB (31, 32), significantly reduced HK-2 cell apoptosis by suppressing the CTSB activity in the LPS-induced HK-2 injury model. Several studies have demonstrated that LMP and CTSB involved various cell death modes, including apoptosis, ferroptosis, and necrosis (12). Zhou et al. reported that the release of CTSB and CTSL from lysosome increased the expression of tBid, active caspase-3, and cytoplastic cytochrome C in oxygen and glucose deprivation model (33). Song et al. reported that LMP occurred in rat proximal tubular cells when exposed to lead. CTSB inhibitor (CA074) and CTSD inhibitor (pepsin A) could significantly inhibit the activation of caspase-3 and cell apoptosis (17). In those kidney injury models, downregulation or inhibition of CTSB activity ameliorated the severity of AKI demonstrated the role of lysosomes or CTSB as a common pathway in S-AKI pathophysiology. It is also possible that LMP and CTSB may prevent S-AKI through other cell death pathways, and our results showed that CTSB significantly triggered apoptosis through mitochondrial apoptosis pathway in LPS-treated cells (30, 31).

Mitochondrial dysfunction has been proven to initiate and accelerate kidney injury (34). One study based on AKI patients and CLP-induced sepsis rat model reported that the mitochondrial dysfunction was closely related to S-AKI (35). CTSB induces mitochondrial outer membrane permeabilization by activating the Bax signaling (36). The released CTSB converts Bid to active truncated Bid (tBid), inducing tBid and Bax mitochondrial translocation and Bax oligomerization, forming pores on the outer mitochondrial membrane. Consequently, mitochondrial intermembrane space proteins are released into the cytoplasm, resulting in cell death.In our study, LPS reduced the mitochondrial membrane potential and activated downstream protein s caspase3/PARP, suggesting that the mitochondrial pathways mediate apoptosis (37, 38).

CA074 is a selective inhibitor of CTSB, has been developed as a promising agent for treatment of different cancers. This study is the first to describe the protective effects of CA074 on AKI by inhibiting the mitochondrial apoptosis pathway. However, further *in vivo* and clinical trials are needed to investigate its effectiveness and safety in the treatment of S-AKI. Inhibition of CTSB may be an effective strategy to treat AKI.

## 5 Conclusion

The finding of this study confirmed that LMP and CTSB contributed to pathogenesis of S-AKI. Moreover, LPS induced LMP and promoted the leakage of CTSB, triggering the mitochondrial apoptosis in HK-2 cells. However, further studies are needed to accelerate the clinical translation of LMP and CTSB. CA074 as an agent inhibit CTSB might be potential drugs for S-AKI treatment.

## Data availability statement

The datasets presented in this study can be found in online repositories. The names of the repository/repositories and accession number(s) can be found below: https://www.iprox.cn/page/project.html?id=IPX0005110000.

## Ethics statement

The animal study was reviewed and approved by Institutional Animal Care and Use Committee, Sun Yat-sen University.

## Author contributions

YW finished the whole experiment and drafted the manuscript. WX, XB and BL performed the animal and cell experiments. XYZ and XMZ performed the analyses, reviewed and edited the draft. XC design the study, and revised the manuscript. All authors contributed to the article and approved the submitted version.
